# Sleep and circadian rhythms in Parkinson’s disease and preclinical models

**DOI:** 10.1186/s13024-021-00504-w

**Published:** 2022-01-09

**Authors:** Jeremy Hunt, Elizabeth J. Coulson, Rajendram Rajnarayanan, Henrik Oster, Aleksandar Videnovic, Oliver Rawashdeh

**Affiliations:** 1 School of Biomedical Sciences, Faculty of Medicine, University of Queensland, Brisbane, Australia; 2 Queensland Brain Institute, University of Queensland, Brisbane, Australia; 3 New York Institute of Technology at Arkansas State University, Jonesboro, AR USA; 4 Institute of Neurobiology, University of Lübeck, Lübeck, Germany; 5 Movement Disorders Unit and Division of Sleep Medicine, Massachusetts General Hospital, Harvard Medical School, Boston, MA USA

**Keywords:** Non-motor symptoms, sleep, circadian, research models, insomnia, Parkinson’s disease, RBD, rapid eye movement, sleep behavior disorder

## Abstract

The use of animals as models of human physiology is, and has been for many years, an indispensable tool for understanding the mechanisms of human disease. In Parkinson’s disease, various mouse models form the cornerstone of these investigations. Early models were developed to reflect the traditional histological features and motor symptoms of Parkinson’s disease. However, it is important that models accurately encompass important facets of the disease to allow for comprehensive mechanistic understanding and translational significance. Circadian rhythm and sleep issues are tightly correlated to Parkinson’s disease, and often arise prior to the presentation of typical motor deficits. It is essential that models used to understand Parkinson’s disease reflect these dysfunctions in circadian rhythms and sleep, both to facilitate investigations into mechanistic interplay between sleep and disease, and to assist in the development of circadian rhythm-facing therapeutic treatments. This review describes the extent to which various genetically- and neurotoxically-induced murine models of Parkinson’s reflect the sleep and circadian abnormalities of Parkinson’s disease observed in the clinic.

## Background

Despite diverse clinical and pathological characteristics, many neurodegenerative diseases share a common fundamental risk factor – ageing. With life expectancy rising globally, the prevalence of these devastating disorders will inevitably increase, imposing a progressively onerous socioeconomic burden on patients, families, and communities. Despite remarkable progress in our understanding of many facets of neurodegenerative diseases, these conditions remain a topic of intense research, and to date, no cures or treatments have been developed that are able to halt their relentless progression.

**Fig. 1:**
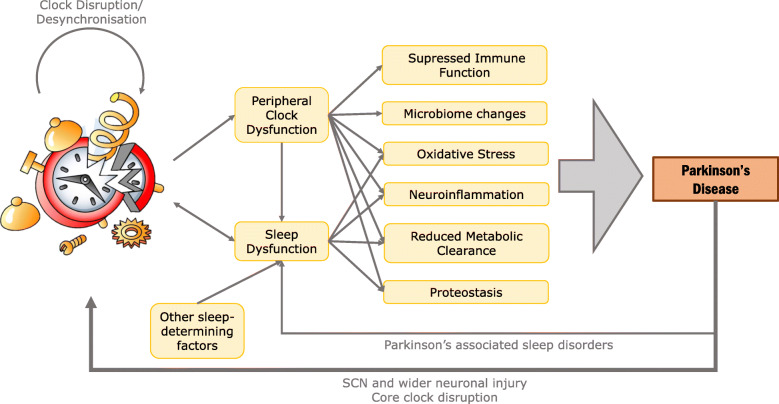
Possible mechanisms of interplay between circadian and sleep dysfunctions and Parkinson’s disease. The suprachiasmatic nucleus (SCN) acts as the central circadian pacemaker, coordinating peripheral clocks, and assisting in the establishment and maintenance of sleep (through process C), which is also contributed by other factors (process S). The SCN uses molecular mechanisms to generate circadian rhythmicity, with zeitgebers (most importantly light) fine-tuning the rhythm. Sleep dysfunction can disrupt these rhythms. Dysfunction of sleep and of peripheral clocks, such as those in microglial and neuronal cells, leads to subsequent changes within and outside of the brain, including neuroinflammation, increased oxidative stress and reduced metabolic clearance. These outcomes have been proposed to increase risk of Parkinson’s disease onset, and to exacerbate Parkinson’s disease progression. Resultant neurodegeneration due to Parkinson’s disease can negatively affect neural pathways, and subsequently desynchronize (or ‘break’) the clock, contributing to a self-perpetuating loop. Parkinson’s disease is also associated with several sleep disorders, negatively impacting sleep and further contributing to the two-way interplay between circadian rhythms and neurodegeneration

It is increasingly well-understood that many neurodegenerative conditions have tight correlations with sleep disorders and circadian dysfunction, most prominently Parkinson’s (PD), Huntington’s (HD) and Alzheimer’s disease (AD) [1–3]. While sleep and circadian rhythms change with age, it is recognized that those with a neurodegenerative disease experience much more severe sleep-wake dysfunction than typical age-related disruptions [4–6]. The sleep disturbances associated with neurodegeneration have significant adverse consequences on both patient and caregiver quality of life; this has increasingly come to the fore following recent advancements in motor symptom management, such as the approval of a number of drugs which can prolong and enhance the action of L-Dopa (e.g., safinamide and opicapone) and improved deep-brain stimulation techniques [7–11]. Further, evidence suggests that sleep disruption is a central aspect of neurodegenerative disorder prodromes [12–14]. There is growing support in the field for the notion that not only is circadian and sleep dysfunction a consequence of neurodegeneration, but that it may also play a causative role, predisposing disease onset and exacerbating disease progression, with circadian dysfunction and neurodegeneration forming a detrimental, self-perpetuating positive-feedback loop (Fig. 1; reviewed extensively in [2, 3, 15–17]). From this, it is clear that consideration of the sleep and circadian facets of disease are of great importance when examining major neurodegenerative diseases.

Murine (rat and mouse) models are arguably the central tool used in investigations of neurodegenerative disease development and progression and thus, considering the increasingly understood importance of sleep as a key facet of these afflictions, it is essential to contemplate the sleep and circadian aspects of such models. Focusing on PD, this review comprehensively categorizes the current understanding of circadian and sleep phenotypes of key murine models of disease (with findings summarized in Table 1). Following an explanation of circadian rhythms and sleep and how they interrelate, we detail the hallmarks of PD, both neuropathological and motor, before describing the sleep and circadian phenotypes of PD patients. Within the different murine models of PD we highlight the reported sleep and circadian phenotypes, and critically evaluate the current findings, comparing these against the present understanding of sleep and circadian disruptions in the clinic. The validity of PD models in light of this comparison is discussed.

**Table 1:** Summary of Findings

Disease Model	How comprehensive is the body of sleep/circadian research in this model?	Does this model reflect human PD-related circadian rhythm changes?	Does this model reflect human PD-related sleep dysfunction?	References
6-OHDA	***	++	+++	[18–36]
MPTP	***	--	---	[37–46]
Rotenone	**	+	+	[47–53]
α-Synuclein Transgenic	**	++	+++	[54–59]
LRRK-G2019S Transgenic	*	N/A	+	[60]
VMAT2-deficient	*	-	--	[61, 62]
MitoPark	*	+++	+++	[63, 64]
Cycad	*	+	N/A	[65]
Paraquat/Maneb	N/A	N/A	N/A	––––
PFF	N/A	N/A	N/A	––––
Other Transgenic models (i.e. DJ-1, PINK)	N/A	N/A	N/A	––––

Table 1**:** Evidence is indicated by – and +, with --- being compelling evidence for a lack of a PD-like phenotype, +++ being compelling evidence of a PD-like phenotype, comprehensiveness of the body of research indicated by asterisks, where *** is very comprehensive, ** requires further studies to corroborate and further confirm, * a very limited number of studies have been relied upon to arrive at the particular conclusion, N/A where no studies have been performed

## Circadian Rhythms and Sleep

The 24-hour day/night cycle is a rhythmic environmental time cue that affects almost all forms of life, most of which have an internal timekeeping mechanism to anticipate and adapt to geophysical time – a so-called ‘circadian clock’. Androsthenes, an admiral who served under Alexander the Great, provided the earliest account of the circadian process, when he described diurnal leaf movements of the tamarind tree in the 4th century BC [66]. However, rigorous scientific evidence for internal circadian clocks only began to emerge in the latter half of the 20^th^ century.

At present, the underlying molecular mechanisms and architecture of the inherent biological circadian clock are well-established (this is discussed with brevity here, but is reviewed in depth elsewhere [67, 68]). The mammalian circadian system is hierarchical, comprised of almost ubiquitous cell-autonomous molecular clocks [69], which are linked, organized and synchronized by a central circadian pacemaker – the suprachiasmatic nucleus (SCN) [70]. At the cellular level, an autoregulatory transcriptional-translational feedback loop (TTFL) generates endogenous rhythms with a period of approximately 24 hours [70]. This regulatory network centers around the positive component of the TTFL, Circadian Locomotor Output Cycles Kaput (CLOCK), along with Neuronal PAS Domain Protein 2 (NPAS2; the paralog of CLOCK in the SCN) and Brain and Muscle ARNT-Like 1 protein (BMAL1, [71, 72]). These transcriptional activators dimerize and bind to *E-box* elements within enhancer/promoter sequences of the negative elements of the TTFL, the *Cryptochrome (CRY1, CRY2)* and *Period (PER1, PER2)* genes to initiate their transcription. The *CRY* and *PER* gene products accumulate, form heterodimers, and negatively act on the function of the CLOCK:BMAL1 complex in the nucleus to repress their own gene expression. E3 ubiquitin ligase complexes are important to reset the system by mediating the degradation of the CRY and PER proteins and, consequently, CLOCK:BMAL1 is able to once again facilitate *CRY* and *PER* transcription. The CLOCK:BMAL1/PER:CRY interaction is further influenced by additional feedback loops, foremost of which involves *RORα/ß/γ* and *REV*-*ERBα/ß,* the rhythmic expression of which is also due to the rhythmic binding of CLOCK:BMAL1 to associated regulatory *E-box* elements. ROR and REV-ERB proteins act as positive and negative regulators of *BMAL1* transcription, respectively, by binding to retinoic acid-related orphan receptor response elements (ROREs) in the *BMAL1* promoter [73]. This additional feedback loop involving both *BMAL1* ROREs is sufficient for generating the rhythm in *BMAL1* expression [74]. The BMAL1-(REV-ERB)-ROR loop, adds robustness and fine-tuning to the molecular circadian pacemaker [73, 75].

These molecular feedback loops generate ubiquitous underlying circadian oscillations in physiology within the brain and peripheral tissues. Peripheral clocks are coordinated and synchronized by the SCN. The SCN master circadian clock consists of approximately 21,000 neurons in C57BL/6J mice [76], between 22,000 and 24,000 neurons (11,400 in males and 11,900 in females, per hemisphere) in Sprague Dawley albino rats [77], and ~83,780 neurons in humans [78], and is located just dorsal to the optic chiasm in the anterior hypothalamus. It receives temporal information in the form of neural input from the retina, commonly referred to as *zeitgebers* (German for ‘time givers’), the most important of which is light [79]. Food availability and temperature cycles are also important *zeitgebers*, particularly for peripheral clocks [71]. In the presence of light, melanopsin-expressing intrinsically photosensitive retinal ganglion cells (ipRGCs) relay photic information to the SCN *via* a monosynaptic pathway. In response to photic stimuli, the SCN communicates time-of-day information by both synaptic and diffusible signals to a host of peripheral oscillators in a variety of brain regions and organs including heart, lung, liver and adrenals [79].

While the circadian clock is strongly associated with the sleep-wake cycle, it has been increasingly implicated in a stunning array of other cellular, (patho-)physiological and behavioral processes, ranging from hormone secretion [80] and metabolism [81], to cardiovascular health [82, 83], cancer susceptibility [84, 85], and neurodegenerative disorders [15, 86]. The latter of these has attracted particular attention, and since the turn of the century researchers have increasingly elucidated undeniable ties between sleep, circadian rhythms and neuropathology.

**Fig. 2:**
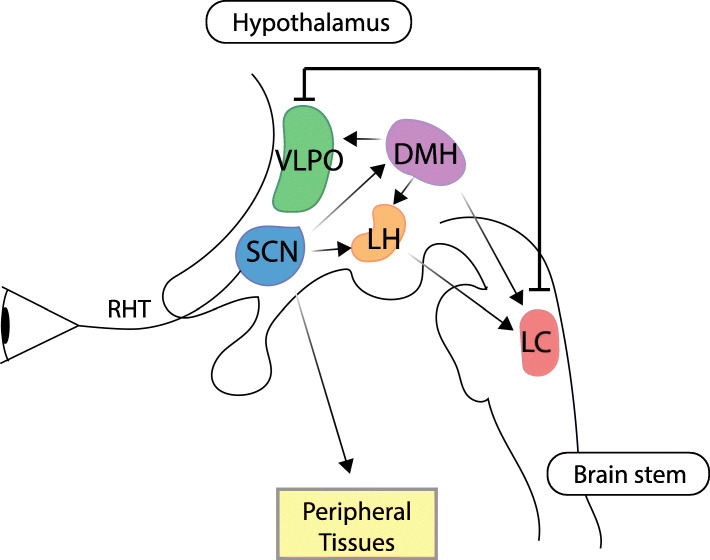
A simplified depiction of mammalian sleep modulation. The suprachiasmatic nucleus (SCN) takes in stimuli from an array of inputs, most notably photic input via the retinohypothalamic tract (RHT). The SCN acts as the central circadian pacemaker, entraining other brain regions and a large array of peripheral tissues, including the liver and gastrointestinal tract, via, both, neural connections and humoral factors. There are direct projections from the SCN to the lateral hypothalamus (LH), which contains orexin neurons, and to the dorsomedial hypothalamus (DMH). The DMH projects broadly to sleep and arousal centers. Mutual inhibition exists between the arousal-promoting locus coeruleus (LC) and the sleep-promoting ventrolateral preoptic area (VLPO) which facilitates the flip/flop between wakefulness and sleep states

Wakefulness and sleep are regulated by multiple brain regions, most prominently the ventrolateral preoptic area (VLPO) of the anterior hypothalamus, the *locus coeruleus* (LC) of the *pons* and the lateral hypothalamus [2] (Fig. 2). In the flip/flop switch model of sleep, mutual inhibition between the VLPO (active during sleep) and monoaminergic nuclei including the LC (active during arousal) allows for the current state to be maintained or, if inputs change, for rapid and complete state transitions [87].

Regarding sleep, there are two main sub-types: rapid eye movement (REM; also referred to as paradoxical sleep, due to associated cortical activation) and non-rapid eye movement sleep (NREM) [88]. These states show distinct physiological signatures, as identified by electromyography (EMG) and electroencephalography (EEG) [89]. NREM sleep is characterized by a variably synchronous cortical EEG – including sleep spindles (SS), K-complexes, and slow waves – accompanied by minimal psychological activity and low muscle tonus. In accordance with the American Academy of Sleep Medicine scoring manual, NREM sleep is divided into three stages: stage 1 is characterized by theta waves with low EEG frequencies (4-8 Hz) and high amplitudes, stage 2 by the periodic appearance of SS (10-15 Hz oscillations which last for a few seconds at most) and K-complexes (single high-amplitude, low-frequency waves which can be considered as the forerunners of delta waves) [90], stage 3 is dominated by delta waves – high-amplitude fluctuations of 0.5-2 Hz [91]. NREM sleep is often referred to as slow-wave sleep (SWS) due to the prevalence of low-frequency waves.

After reaching stage 3 of NREM sleep, the sequence reverses itself and a period of rapid eye movement sleep, or REM sleep, ensues. REM sleep is characterized by low-voltage, high-frequency (10-60 Hz) activity similar to the EEG activity of individuals who are awake. Aside from high-frequency EEG activity, REM sleep is defined by skeletal muscle hypotonia, vital sign instability and rapid horizontal eye movements. During sleep, the brain cycles between NREM and REM sleep; in adults, approximately 80% of total sleep time is spent in NREM states [92].

Two separate processes influence the 24-hour sleep-wake cycle: ‘sleep propensity’ (process S; also referred to as ‘sleep drive’), a homeostatic component determined by the immediate history of sleep, and circadian rhythms (process C), driven by the endogenous biological clock [93]. The constant interaction between these two processes in humans enables for consolidated sleep and wakefulness to be confined to nighttime and daytime, respectively [94]. The homeostatic process accumulates during wakefulness, eliciting sleep as it reaches an upper boundary and dissipates during the sleep state, triggering wakefulness as it reaches a lower boundary. To counteract the diminishing homeostatic need for sleep towards the conclusion of the sleep cycle, the circadian propensity for sleep increases during the sleep state, ensuring a consolidated sleep bout. The central circadian system initiates the secretion of melatonin, a key sleep promoting hormone, from the pineal gland approximately 2 hours prior to sleep onset [95, 96]. A recently introduced unified model of sleep-wake regulation by Lazarus and colleagues proposes that the sleep state is regulated by arousal when an organism must consolidate wakefulness in response to environmental changes [97]. According to this model, the circadian and hypothalamic feeding systems have indirect influences on sleep by driving internally generated arousal (e.g., circadian regulation of the motivation to forage). This model is based on the new roles of adenosine receptors in modulating different aspects of sleep. Adenosine receptors A_2A_ (A_2A_Rs) appear to promote sleep-suppressing arousal [98], whereas sleep need and the response to sleep deprivation are mediated by A_1_Rs [99]. These receptors therefore play crucial roles in the function of sleep. Interestingly, A_2A_Rs are also densely expressed on striatopallidal neurons (e.g., in the globus pallidus externus; GPe) in the basal ganglia (BG), in which dopamine D_2_ receptors are coexpressed with the A_2A_Rs and contribute to the control of activities that require wakefulness (e.g., locomotion and motivation) [100, 101]. The striatopallidal neurons also operate with dopamine D_1_ receptor-bearing striatonigral neurons in the direct pathway of the BG. Prototypically and arkypallidal GPe neurons fire at inversed phases both during sleep and active states, and this difference is exacerbated in the setting of dopaminergic denervation [102]. Abilities to maintain arousal are compromised under low dopamine conditions, as in Parkinson's disease [103–105].

Since this review concerns mouse and rat models, it is necessary to briefly consider aspects of both convergence and divergence between human and animal sleep and circadian rhythms. Features of sleep, including duration, diurnal timing and cyclical organization have been extensively categorized in most murine strains [106], although just as sleep patterns vary between human populations and, indeed, between individuals, even inbred strains of laboratory mice show variation in these properties of sleep [107].

While in humans, the primary diurnal sleep phase is at night, for rats and mice, it occurs during the light phase [108]. Further, rodents exhibit sleep bouts across both the day and nighttime (in rats the bouts are between 10 and 14 minutes, in mice, 2 and 4 minutes), otherwise known as a polyphasic sleep pattern. Humans usually show monophasic sleep patterns with sleep bouts of 6-8 hours in length (although sleep patterns may be diphasic). Within this consolidated block of sleep, humans typically pass through 4 to 6 cycles of NREM and REM sleep, whereas in rodents, cycles are substantially shorter and occur periodically across the 24-hour day [109].

Age-dependent changes in sleep architecture and the temporal distribution of sleep in mice and rats share significant similarities with age-dependent changes in humans. In humans, age-related sleep changes include increased daytime napping, advanced sleep timing, decreased nocturnal sleep duration and fragmentation of nocturnal sleep [110, 111]. Further, mechanisms of sleep homeostasis and the circadian system become less robust with normal aging [112]. Increased sleep fragmentation is seen in older mice [113], as well as a larger proportion of total sleep during the active phase [114]; an increased inability to maintain wakefulness and sleep and age-related EEG spectral profile alterations have been well characterized in rats [115].

## Parkinson’s Disease

### Background

The first formal description of Parkinson’s disease came in the 1817 “Essay on the Shaking Palsy” in which the cardinal and distinguishing symptoms of what was originally termed as *paralysis agitans* were characterized by the disorder’s namesake, James Parkinson [116]. Today, over 200 years later, PD is the second most prevalent neurodegenerative disorder globally and, with rising life expectancy across the globe, an accompanying increase in disease burden is inevitable [117, 118].

Neuroanatomically, PD is characterized by widespread subcortical structure degeneration, particularly in the dopaminergic neurons of the *substantia nigra pars compacta* (SNpc), which results in a significant decrease in the dopamine (DA) content of the striatum. This degeneration is accompanied by inclusions in specific nerve cells, known as Lewy bodies, which were first detailed by Friedrich Lewy in 191 2 [119, 120]. The constitution of these inclusions eluded researchers until a single missense mutation in the *α-synuclein* (*SNCA*) gene was identified as the cause of an autosomal-dominant form of PD [121]. α-synuclein protein was subsequently identified and confirmed as the major component of the characteristic fibrils which form Lewy bodies and Lewy neurites [122]. Today, the role of α-synuclein in PD is still contended, the debate as to potential neuroprotective or toxic roles is well-considered [123], however there is overwhelming evidence that Lewy pathology is unlikely to be benign [124], and there is increasing acceptance in the field that PD can be classified as a prionoid disorder, driven by the spread, seeding and aggregation of misfolded α-synuclein [125, 126]. Most commonly, PD arises idiopathically, however familial forms of the disease can occur, most frequently due to inherited mutations in *SNCA*, *LRRK2* (leucin-rich repeat kinase 2), *PARK7* (parkinson protein 7), *PINK1* (PTEN-induced kinase 1), or *PRKN* (parkin) genes.

PD has a clinical presentation of progressively worsening bradykinesia, typically accompanied by either gait disturbance, resting tremor or rigidity [125, 127, 128], however it is commonly accepted that PD-related neurodegeneration can begin several decades before motor symptom onset [129, 130]. A range of non-motor symptoms, some of which arise prodromal to clinical onset of disease [129, 130], are strong correlates of PD including autonomic dysfunction [131–134], neuropsychiatric symptoms [135–138], dementia [139, 140], sensory symptoms [141, 142] including loss of olfaction (which commonly predates motor symptoms )[143] and sleep and circadian rhythm dysfunction [144–146]. In the following section we will summarize the sleep and circadian phenotypes in PD – for a more in-depth analysis of this see these recent reviews [3, 144, 147–149].

### Circadian Rhythms

Parkinson’s seminal categorization highlighted that patients were often ‘constantly sleepy’ and ‘exhausted ’[116]; and a recent systematic literature review found that up to 80 % of PD patients suffer from sleep-wake disturbances [150]. Circadian alterations in PD patients have been investigated by an array of research groups using an assortment of circadian measures. Nocturnal hypertension is near-ubiquitous in PD [151–153], and often patients exhibit a reversed circadian rhythm in blood pressure, with nocturnal blood pressure greater or equal to daytime levels [151]. PD patients have also been observed to have disruptions in circadian thermoregulation, with significant reductions in the mesor (the rhythm-adjusted mean level of a response variable around which a wave function oscillates) of the core body temperature (CBT) rhythm and dampened CBT rhythms, both of which are strongly correlated with severe sleep issues [154]. Endocrine parameters are considered as major circadian indicators, and disruptions in hormone rhythms have been highlighted in PD. In particular, PD patients do not exhibit significant daytime-dependent variation in plasma melatonin concentrations [155, 156]. Further, the diurnal cortisol secretory profile, specifically, its peak, is compressed in PD patients [155, 157]. It should be noted that some other circadian-modulated hormones are seemingly unaffected in PD, including growth hormone, thyroid-stimulating hormone, prolactin, as well as certain fat tissue-associated hormones [158, 159].

Metabolic abnormalities, most notably body weight loss, have been widely reported in PD patients [160, 161] associated with rapid disease progression and poor clinical outcomes [162]. Body weight is dependent on the interplay of energy intake and energy expenditure, the latter of which is regulated by peripheral circadian physiology in cells throughout the body [163]. Compromised circadian modulation prospectively underlies dysregulation of energy expenditure in PD, especially in temporal rhythms of energy metabolism [54].

In a recent review, Leng and colleagues provided strong evidence for Parkinson’s-associated circadian disruptions based on an array of circadian-focused PD investigations [3]. Circadian related sleep-wake disturbances were clearly observed in five out of six studies, including reported changes in sleep timing [154, 155, 164] and excessive daytime sleepiness [4, 155, 165].

Studies investigating changes in the molecular clock mechanism have highlighted altered *BMAL1* mRNA expression in Parkinson’s patients. Compared to healthy individuals, nighttime *BMAL1* expression levels have been observed to be significantly lower in PD-patient cohorts (which included patients who had previously received, or were currently receiving, medication to treat PD as well as unmedicated individuals – known as drug-naïve patients) [18, 166]. Decreased *BMAL1* expression levels were observed both in drug-naïve and medicated patients suggesting the change was independent of drug treatment (medicated patients were receiving a mean dose of L-Dopa equivalent to 310±58.6 mg). The same study also reported that *BMAL1* levels correlated with PD severity [166]. Increased expression of *PER2* and *REV-ERBα* during the early morning has also been observed in PD patients [155]. A secondary finding of this study was a dampened *BMAL1* expression profile in those with PD compared to healthy controls.

It is known that dopamine acts as a circadian clock input, enhancing the transcription of the CLOCK:BMAL-1 complex *via* the activation of dopamine receptors in the nervous system (including in the SCN) [167–169], however much more extensive mechanistic studies are required to completely characterize clock gene expression and potential regulatory mechanisms in PD.

It has been noted extensively in reviews on this theme that dopaminergic treatment can affect the circadian rhythms of Parkinson’s patients [3, 144] (e.g., it has been established that treatment with L-Dopa can phase advance the melatonin rhythm [170, 171]). Clarification on circadian alterations in *de-novo* PD will require a clear separation between drug-receiving and drug-naïve PD groups. The evidence currently available from drug-naïve PD patient studies shows that circadian disruptions manifest as a reduction in the amplitude of circadian rhythms, without a significant phase shift or change in period, a pattern of circadian alterations known as ‘chronodegeneration ’[3, 4, 172].

### Sleep

Sleep issues accompany PD with near ubiquity [148, 173–179] and have significant negative repercussions on, both, patient and caregiver quality of life. It has been illustrated in a number of studies that PD patients experience marked changes in sleep architecture, although findings are not entirely consistent [144]. Macro-structure changes in sleep architecture manifest most commonly as sleep fragmentation and relative increases in superficial sleep [180–185], and altered sleep micro-structure (e.g., the loss of sleep stage integrity) has been observed in a number of studies, albeit these had small sample sizes [186–189]. Using a state space model, which quantitatively represents sleep EEG as a trajectory in 2-dimensional space (where state space velocity correlates to sleep state instability), it has been noted that, in PD patients, transitions between distinct stable sleep stages are much slower (a phenomena termed bradysomnia )[190]. There does exist some heterogeneity within PD patient cohorts, and while the changes to sleep structure defined here are typical of PD, the extent to which all are observed differs by patient and progresses across the course of disease [191–193].

REM sleep has been a particular focus of PD sleep disorder studies. Deficiencies in REM sleep are commonly associated with disease including diminished REM sleep [155, 194, 195] and increased latency to reach the REM stage [195, 196], although dialogue still exists surrounding the extent of these changes [192, 197]. Further, the REM sleep EEG is often typified by a sustained increase in high-theta/alpha frequencies (7.8-10.5 Hz) in the beginning of the sleep period in Parkinson’s patients [198], although the ubiquity of these changes is debated [188]. Diederich and colleagues’ seminal retrospective study concluded that sleep ‘destructuring’, defined as longer sleep latency, less time in deep sleep and REM, and reduced sleep efficiency, is aligned to the progression of PD independent of other major disease parameters including medication [199].

REM sleep stage disruptions contribute to one of the most common PD-associated sleep disorders, REM sleep behavior disorder (RBD), which is characterized by complex motor behaviors and loss of muscle atonia during REM sleep. In the general population, the prevalence of RBD is approximately 0.04–0.05 % [200]. In sharp contrast, RBD presents in up to 47 % of PD cases [201]. The cumulative risk of neurodegenerative disease 14 years post-RBD onset exceeds 90 % [202, 203] and as such RBD is considered a prodromal marker of neurodegeneration, most strongly associated with synucleinopathies [204]. RBD has the greatest predictive power and specificity of any prodromal marker of PD [205], and is also a predictor of cognitive decline and motor symptom progression [206].

The *locus coeruleus* plays a crucial role in the PD prodrome; dysfunction and degeneration of noradrenergic neurons in the LC are associated with RBD, as well as reduced arousal [207]. It has been proposed that the LC therefore represents an important area to study in experimental animal models to further characterize the PD prodrome [208].

Restless legs syndrome (RLS), another co-morbid sleep-wake disorder characterized by an irrepressible compulsion for limb movement, frequently accompanied by unpleasant limb sensation, is observed in as many as 20 % of Parkinson’s patients [209]; it is observed in 3.9–14.3% of the general population [210]. This urge usually arises after extended periods of inactivity and is typically alleviated temporarily by movement of the legs. Notably, it has been suggested that dopaminergic drug therapy as opposed to the disease is the most significant contributor of RLS [211, 212]. Although some studies on *de-novo* patients have demonstrated a relationship between the two disorders [213], it has been proposed that PD is a risk factor for leg motor restlessness, often incorrectly diagnosed as RLS [144, 214].

Insomnia, the inability to initiate and/or maintain sleep (often described as fragmented sleep), or the perception of the inability to sleep, is the most common sleep disorder reported in PD with sleep maintenance problems affecting up to 85 % of patients [215]. Over the course of PD, sleep initiation problems become less frequent, whereas sleep maintenance problems become more prevalent [216]. Interestingly, insomnia is often an attribute of Parkinson’s cases where motor symptoms are improved upon awakening in the morning. The underlying mechanism of this observation, however, is unclear [217].

The last of the PD-related sleep disorders to be described here is excessive daytime sleepiness (EDS), the pathologically enhanced propensity to fall asleep during daytime. There exists a significant discrepancy in the described epidemiology of this disorder in PD, with one review suggesting a prevalence range of 15–76% in PD patients [144]. This large variance in EDS incidence concurrent to PD may be explained by inconsistencies in both subjective and objective assessments. Despite this, EDS prevalence is widely understood to increase with disease progression [218]. While EDS has been reported in drug-naïve patients [219], it occurs with much higher frequency in medicated patients [220, 221]. Interestingly, a recent study, which included both treated and untreated PD patients, found no relationship between EDS and nocturnal sleep disturbances, suggesting that in PD daytime sleepiness may be an independent symptom unrelated to sleep quality [222].

## Circadian and Sleep Phenotypes of Murine Models of PD

### 6-OHDA Model

6-hydroxydopamine (6-OHDA) a structural analogue of dopamine and noradrenaline, undergoes rapid auto-oxidation to exert toxic effects on catecholaminergic neurons [19]. The first murine 6-OHDA neurotoxic model exhibiting akinesia with a very high mortality rate was developed in 1968 and is comprised of bilateral toxin injection into the SNpc [20]. Injections of 6-OHDA promote the uptake of the neurotoxin *via* membrane-bound dopamine and noradrenergic transporters. The subsequent intracellular accumulation of 6-OHDA inhibits the mitochondrial respiratory chain complex I, generating reactive oxygen species (ROS) that cause oxidative stress-related cytotoxicity [21]. 6-OHDA is typically injected stereotactically into particular brain regions to bypass the blood–brain barrier and to target certain regions. Commonly utilized models include both unilateral [223, 224] and bilateral [22] intra-striatal injections which can simulate the nigrostriatal damage observed in PD by causing progressive loss of dopaminergic neurons of the SNpc. The extent of neurodegeneration is dependent upon the site of lesion and dose of 6-OHDA administered [23]. Like many of the neurotoxic models of PD, the acute neurodegenerative property of the 6-OHDA model is an imperfect fit of the progressive, age-dependent degeneration seen in PD. Additionally, Lewy bodies are not present in this model [19].

#### Circadian Rhythms

Physiologically, 6-OHDA lesioned rodents have been shown to exhibit circadian perturbations. One of the early investigations into circadian alterations mediated by dopamine depletion in 6-OHDA lesioned rats identified a significant decrease in the mesor and a phase advance of heart rate and temperature rhythms – two key circadian clock outputs [24, 225]. Transient thermoregulatory dysfunction in 6-OHDA rats in the week following lesioning was described in an independent publication, however, later on the animals exhibited recovery of these metrics to levels comparable to controls [25]. While the former studies utilized a dual bilateral injection into the anterior and posterior striatum [26], the latter employed a unilateral injection into the medial forebrain bundle [27], demonstrating the heterogeneity of findings contingent on the methodology. Bilateral injection models are more likely to exhibit a more severe phenotype, which is reflected by these findings.

Further physiological alterations in the 6-OHDA model have been observed in cardiovascular circadian markers, suggesting subtle autonomic dysfunction. The dip in blood pressure and heart rate that occurs during the light (i.e., resting) period in rats – analogous to the nocturnal dip in healthy humans – is significantly disturbed following 6-OHDA injection into the ventral tegmental area (VTA) [28]. Rats with unilateral medial forebrain bundle lesions display decreased nocturnal heart rates compared with sham controls [25] and suppression of arterial blood pressure and heart rate increases during REM sleep in ventral tegmental area-injected rats [29]. Cardiovascular impairments, including a decrease in baseline mean arterial pressure and heart rate, have been observed by Ariza and colleagues in 6-OHDA lesioned murine models [30]. Spectral analysis of systolic arterial pressure in the bilateral 6-OHDA lesioned substantia nigra suggests a possible decreased sympathetic activity. These results support findings in humans that show that the sympathetic nervous system activity is attenuated in patients with PD and REM sleep behavioral disorder when the disease progresses to PD [31].

Locomotor activity of the 6-OHDA murine model has been studied by several groups to analyze circadian behavioral changes. Isobe and Nishino pioneered this investigation, finding significant hypoactivity in lesioned rats, especially during the dark phase (active period), without phase-shifting [32]. In addition, under free-running conditions, the endogenous circadian periods of both blind (unable to entrain their circadian system to photic information) and sighted 6-OHDA lesioned rats increased relative to sham controls. These findings have since been supported by a multitude of publications in both bilateral and unilateral lesioned rat and mouse models [24, 33–35, 226, 227]. A more recent study of 6-OHDA treated mice identified a persistence of 24-h rhythmicity in sham mice, and a loss of this endogenous rhythmicity in lesioned mice, with a fragmentation of the activity pattern of the lesioned mice [35]. Interestingly, the authors of this study were able to use a histamine H3 receptor antagonist, thioperamide, to rescue the normal sleep/wake phenotype, suggesting that the H3 receptor represents a potential target for multifunctional treatment of PD-associated sleep/wake disturbance and cognitive impairment.

The molecular basis of circadian rhythmicity has been an area of increased recent interest in the 6-OHDA model, especially in light of findings that PD patients often have disruptions in the levels of circadian proteins [18]. A pair of studies have established that 6-OHDA lesions affect PER2 protein expression in the rat; in animals with unilateral medial forebrain bundle injections, the neurotoxin caused significant blunting of peak *PER2* transcription and expression levels in the dorsal striatum of the injected side as well as in catecholaminergic fiber-innervated areas such as periventricular hypothalamic nucleus, but not in the SCN or on the lesion-contralateral side [33, 36]. It was uncovered that unilateral 6-OHDA lesion selectively reduces the normal morning peak of PER1 expression in the lesion-ipsilateral dorsal striatum without affecting ipsilateral SCN PER1 expression [36]. Further, it has been shown that striatum mRNA levels of *BMAL1*, *PER2*, and *CLOCK* are significantly decreased in 6-OHDA-lesioned rats, as well as increased acetylation of BMAL1, potentially mediated by SIRT1 (which deacetylates BMAL1 and PER2) [228]. In *REV-ERBα* knockout mice with unilateral 6-OHDA induced lesions in the dorsal striatum, neurodegeneration is exacerbated, mirroring the clinical findings that circadian dysfunction can predispose to PD [229]. In summary, the observations that clock gene expression is altered following 6-OHDA-induced neurodegeneration, suggests that this preclinical PD model could be utilized to explore the relationship between PD-like neurodegeneration and changes to circadian clock and clock-regulated gene expression.

Jiang and colleagues recently identified a PD-associated co-expressed network module named *Mediumpurple2*, which was strongly associated with sleep fragmentation phenotypes [230]. It was found that this gene network was robustly upregulated across several genetic and neurotoxic murine models of PD and highlighted significantly elevated expression of *Mediumpurple2* module genes in the striatal *DRD1*^*+/+*^ medium spiny neurons of 6-OHDA-lesioned mice. On the other hand, *Mediumpurple2* module genes were downregulated in *DRD2*^*+/+*^ neurons.

Collectively, the murine 6-OHDA models broadly recapitulate the circadian dysfunction seen in clinical PD (Table 1). A limitation of the model, however, is that often changes to circadian rhythmicity are of a short term, or transient, nature; partial recovery of the circadian phenotype occurs a few weeks after administration of the neurotoxin. This is to be expected, given the model mechanism – acute damage to neurons at time of injection – however it limits investigations into progressive circadian degeneration, as seen in PD patients. As such we suggest that the 6-OHDA model provides a strong model as to investigate how PD-like neurodegeneration contributes to circadian arrhythmicity, even if it may not be able to be employed to investigate progressive circadian dysfunction.

#### Sleep

A number of the aforementioned studies which quantified 24-hour locomotor activity utilized these data to calculate non-qualitative sleep metrics. Rats with targeted 6-OHDA lesions of the VTA, were more active during the resting period and showed increased immobility-defined sleep during the active phase, closely resembling the night-time insomnia and day-time somnolence, respectively, of Parkinson’s patients [32, 226]. Rats lesioned with 6-OHDA in the medial forebrain bundle of the striato-nigral system, on the other hand, displayed a phenotype suggested to parallel the nocturnal akinesia in untreated PD patients^154^. Taken together, these findings suggest that the sleep phenotype is highly dependent on the lesion site, and that the lesions affecting mainly the VTA (and the A10 cell group) lead to sleep-wake disturbance-related sleep changes, whereas lesions in the medial forebrain bundle (targeting the A9 cell group) primarily affect motor-related sleep alterations.

More comprehensive sleep-focused investigations in VTA-targeted 6-OHDA-lesioned rodents focused on EEG and EMG monitoring. Sei and colleagues noted suppression of the frequency of EEG theta rhythm during both wakefulness and REM sleep, postulating that midbrain dopaminergic neurons are involved in the hippocampal theta activity in rats [29]. Another group used similar methods and found that total NREM and REM sleep duration was significantly decreased during the sleep-phase and increased during the wake-phase in 6-OHDA-treated animals, results that align with commonly accepted understandings of REM sleep deterioration in PD [28].

In a recent investigation into sleep alterations in the 6-OHDA model, Ciric and colleagues saw significant sleep microarchitecture abnormality in unilateral SNpc lesioned rats [231]. Augmented theta amplitude across sleep states was seen in the motor cortex and in the hippocampus. Moreover, significant sleep spindle (SS) alterations and high voltage spindles (HVS; 5-13 Hz) were observed: the unilateral SNpc lesion increased the density of the SS during NREM sleep in both the motor cortex and the hippocampus. While debate surrounds the function of HVS (being analogous to the human mu rhythms) [232], evidence suggests that HVS and SS dynamics are regulated by thalamo-cortical circuits and that abnormal dynamics reflect the dysfunction of these circuits [233]. Further, EEG oscillation synchronization was significantly impacted in the 6-OHDA model. When taken together, these findings suggest that 6-OHDA rats have significant changes in sleep microstructure, comparable to changes observed in the sleep microarchitecture of human PD patients. Further, observed severe sleep fragmentation is analogous to an insomnia-like behavioral phenotype, which provides merit for the ability of this model to reflect PD-concomitant sleep degradation.

Urethane anesthesia can be employed in the rat to mimic fluctuations between REM and NREM sleep and has been used to analyze sleep state transitions in the 6-OHDA model [234]. In animals in which clear urethane-induced sleep-like states were observed using fMRI, there was significantly lower functional connectivity between brain regions during REM sleep. This reflects a role of the nigrostriatal dopaminergic pathway in REM sleep, and importantly provides further mechanistic understanding of sleep disturbance in PD patients.

These studies collectively suggest that the preclinical 6-OHDA lesion model suitably reflects the disease state sleep phenotype seen in human PD, although given the model mechanism, it does not reflect the sleep change progression that often correlates with disease progression (see Table 1). Notably, the presently recorded changes in sleep parameters in the 6-OHDA model do not recapitulate all facets of human-PD sleep alterations. There is, however, compelling evidence that the location of the 6-OHDA lesion influences the exact sleep phenotype observed, as briefly discussed above, which should be considered in future investigations utilizing the 6-OHDA model. Again, it suggests a compelling case for a role of 6-OHDA as a model in which to investigate links between neurodegeneration and sleep dysfunction in PD.

### MPTP Model

In the early 1980s, a series of intravenous drug users were admitted to Californian hospitals with severe Parkinsonian-like symptoms; it was discovered that the symptoms were caused by 1-methyl-4-phenyl-1,2,3,6-tetrahydropyridine (MPTP), which had contaminated a batch of synthetic meperidine [235, 236]. This discovery led Langston and colleagues to recognize the potential of utilizing this neurotoxin to create a PD animal model [237, 238].

Due to its lipophilic properties MPTP is able to transverse the blood–brain barrier, where it is often taken up by astrocytes [37]. Astrocytic monoamine oxidase-B metabolizes MPTP producing the active toxic cation 1-methyl-4-phenylpyridinium (MPP+). When MPP+ is released into the extracellular space it is taken up by dopaminergic neurons, where it induces neurotoxicity by inhibiting the mitochondrial respiratory complex I causing ATP depletion and oxidative stress [38, 39].

The conventional MPTP model involves acute or subacute administration. This acute MPTP administration produces rapid cell death and extensive lesioning of dopaminergic SNpc neurons – these lesions are highly variable and often reversible [40]. At present, chronic low neurotoxic doses are used to better replicate the time-dependent progression of PD [41], however, due to the rapid turnover of MPTP to MPP+, such chronic treatments represent at best serial acute insults [42]. Reports vary as to the extent that mice treated with MPTP display behavioral deficits reminiscent of PD, however alterations in motor movement are typically observed [42]. Again, a key shortcoming of this model is the lack of reliable Lewy body formation, although α-synuclein inclusions are observed in non-human primate MPTP models [43].

#### Circadian Rhythms

A longitudinal study into the circadian features of MPTP-treated mice established that the circadian dysfunction typically associated with PD is poorly reflected in this model [44]. The fundamental properties of the circadian system including endogenous free-running rhythms and entrainment remain intact in both the chronic and acute MPTP models. Furthermore, age-related deterioration in circadian rhythms and response to time cues were not exacerbated by the neurotoxin treatment protocol, which questions the validity of the MPTP model in replicating the non-motor aspects of PD. Hayashi *et al.* also found that MPTP-treatment in mice significantly lowers locomotor activity during the active phase, and promotes arrhythmicity in core body temperature patterns and the dampening of circadian clock gene expression in the SCN [239].

At a molecular level, SCN clock gene expression profiles, although rhythmic in MPTP treated mice, for both the positive and negative components of the TTFL were unaltered [239]. Further investigations into clock gene expression in the MPTP model are required to confirm and further validate the SCN clock gene expression profiles in this model.

Based on these investigations into circadian dysfunction in murine MPTP models, it is suggested that the model does not reproduce the circadian dysfunction typical of human PD and questions the model’s suitability for Parkinson’s research, particularly in understanding the connection between PD-like neurodegeneration and the presentation of non-motor symptoms (see also Table 1).

As previously discussed, it has been postulated that circadian disruption and sleep dysfunction can exacerbate both PD symptomology and progression and findings in the MPTP model support this. In MPTP-treated mice chronic circadian disruption aggravates MPTP-induced motor deficits and further impairs motor skill learning [240]; this suggests that the MPTP model could be employed to investigate the role of sleep and circadian dysfunction in a model of neurodegeneration, even if it is a less than perfect model of PD.

#### Sleep

Subsequent to neurotoxic treatment, the sleep phenotype of MPTP animals is modified, however a multitude of publications have asserted that these alterations are not reflective of sleep changes experienced by humans across the course of PD. Compared with controls, MPTP-treated mice present with changes in sleep architecture throughout the nychthemeral period, with longer wakefulness and paradoxical sleep episodes and an increase in the amount of paradoxical sleep [241]. Furthermore, a long-term analysis of sleep alterations found no correlation between dopaminergic neuronal loss and paradoxical sleep alterations in mice subject to a subchronic MPTP administration protocol (five once-daily injections of 25 mg/kg )[45]. Further, no significant differences were observed in any additional sleep parameters despite considerable dopaminergic neuronal loss in the treated mice, as corroborated by later studies [46, 242, 243]. These findings can be explained by evidence that MPTP-induced alterations are transitory, from a number of publications documenting the histological and behavioral recovery processes in MPTP [244–246]. Further, it was suggested that other neurotransmission systems may be involved in paradoxical sleep variations in MPTP mice [45].

Extending from this, Lima *et al.* found that while dopaminergic neurons present in the SNpc possess a fundamental function in the regulation of sleep processes, particularly in promoting REM sleep, MPTP rats did not display a sleep disruption phenotype that replicated Parkinson’s [247]. Despite not being the focus of their studies, two of the wide-ranging circadian investigations into the MPTP model discussed in the above section acknowledged a lack of sleep changes in the treated animals [44, 47].

Collectively, the evidence suggests Parkinsonian-esque sleep disruption does not exist for the MPTP model, raising further doubts as to its applicability and validity as a robust and comprehensive model of PD, specifically for the study of the non-motor aspects of the disease (summarized in Table 1).

### Rotenone Model

The organic pesticide, rotenone, was identified as a potential neurotoxic agent due to its function as a potent mitochondrial respiratory complex I inhibitor. Highly hydrophobic, it is able to easily cross the blood-brain barrier, and once in cells causes mitochondrial dysfunction and reactive oxygen species production. Rotenone exerts its effects in a dopamine transporter-independent fashion, thereby eliciting mitochondrial dysfunction in non-dopaminergic systems as well. The modern rotenone model was pioneered by Betarbet and colleagues who subcutaneously implanted osmotic mini-pumps to deliver continuous intravenous infusion of low-dose rotenone in Lewis rats [48]. While its mechanism of toxicity is not specific to nigrostriatal dopaminergic neurons, the neurotoxic effects are predominantly dopamine neuron-selective [49]; this is likely in part due to higher inherent vulnerability of SNpc dopaminergic neurons to mitochondrial damage [50]. High variability in intra- and inter-species sensitivity coupled with a potential induction of widespread systemic toxicity complicates the use of rotenone to model PD [51]. Revised rotenone model protocols have been developed, which aim to produce more consistent nigrostriatal pathway lesion and α-synuclein and ubiquitin-positive neural inclusions [248]. Nonetheless, the model has not been widely adopted, due to the existence of more established neurotoxic models coupled with the fact that despite efforts to mitigate the issues highlighted above, revised protocols remain beset, albeit to a lesser extent [52, 248].

#### Circadian Rhythms

The first investigation into the effect on the circadian rhythms of rotenone administration in rodents was performed by Lax *et al.* who found that rotenone treatment significantly dampens the amplitude of the CBT rhythm [53]. Additional analysis also revealed a reduced mesor and amplitude of the sleep/wake rhythm in rotenone-treated rats, which correlated to the severity of motor impairment, suggesting the degeneration of nigrostriatal dopaminergic neurons as the cause of this finding.

Alterations and disruptions in daily rhythms of serotonin metabolites, most notably in the amplitude of circadian clock gene expression rhythms (*Per1*, *Cry1* and *Bmal1*), decreases in the daily pulse of *Per1*, *Per2, Cry1, Cry2* and *Bmal1* as well as significant changes to pairwise correlation during both light and dark phases, has been observed in the rotenone model [249]. A decrease in the expression of several clock genes has been reported under conditions where low-grade neuroinflammation (caused by lipopolysaccharide injection) aggravated rotenone-engendered neurodegeneration [250]. Notably, this study only analyzed clock gene expression at a single time-point, as such one cannot distinguish between a decrease in clock gene expression due to a shift in the expression rhythm and the direct effect of rotenone on clock gene expression. However, the observation warrants further investigation into changes to the gene expression across the full 24-hour period.

Of interest is a recent study in which rotenone-administered rats were subjected to chronic sleep restriction. These animals showed stronger perturbations in metabolic and biochemical rhythms when compared to sleep-restricted untreated and non-sleep restricted rotenone-treated animals. This finding provides attestation to the widely held theory that in humans, PD symptoms can be exacerbated by sleep dysfunction [251].

There exists some promise that the rotenone model credibly models aspects of circadian dysfunction seen in PD, however at present there are significant gaps in the characterization of the circadian phenotype of this animal model and, as such, further investigations are required (Table 1).

#### Sleep

Rotenone administration was reported to produce progressive deterioration of sleep in rats: reduced duration of NREM sleep, decreased frequency of REM sleep and an increased number of awakenings. Surprisingly the same results characterizing sleep were obtained in vehicle treated animals (vehicle: dimethyl sulfoxide (DMSO) and polyethylene glycol (PEG) (1,1) )[252]. As would be expected, vehicle animals failed to exhibit the characteristic neuropathology of the model; it was concluded then that sleep changes seen in both the control and treated rats were independent of dopaminergic cell loss. The validity of the rotenone rat PD model was therefore called into question.

It has been shown that while intraperitoneal administration of 5% and 10% DMSO solutions in male rats have no significant effect on sleep/wake parameters and EEG power spectral analyses, 15% and 20% solutions of DMSO decrease the mean episode duration of deep slow-wave sleep and increase light slow-wave sleep [55]. However, no discrepancies between the sleep behaviors of saline treated rats and DMSO/PEG treated rats were observed in two other studies [56, 57]. Ideally, lower concentrations of DMSO, or other vehicle solutions, should be employed if utilizing rotenone as a neurotoxin-induced model of PD, and saline treated controls are essential to validate results.

In the two studies where the saline and vehicle controls had consistent findings, the authors surmised that modified sleep parameters in rotenone-treated animals solely resulted from rotenone neurotoxicity. Yi *et al.* found rotenone-treated rats develop similar sleep disturbances to those clinically correlated with PD; increases in slow-wave sleep and REM sleep during the animals’ active phase and decreases in slow-wave sleep in the rest phase replicate the non-motor symptoms of excessive daytime sleepiness and insomnia, respectively [56]. Targa and colleagues, while investigating PD sleep regulation circuitry, found pronounced REM and NREM sleep changes resulting from a rotenone-induced striatal lesion, however cautioned readers about the applicability of this finding to human PD; the sleep phenotype did not fully recreate that commonly seen in patients [57]. In both studies, DMSO/PEG vehicle solution was employed, however no discrepancies between the sleep behaviors of pyrogen-free saline (PFS) treated rats and DMSO/PEG treated rats were observed, suggesting modified sleep parameters solely resulted from rotenone neurotoxicity. A word of caution about both studies; although Yi *et al.* reported no effect of 50 % DMSO / 50 % PEG on sleep parameters compared to PFS, the actual presented data only compared PFS with rotenone treated groups. As for the study by Targa *et al.*, no information was provided about the percentage of DMSO used to dissolve rotenone.

At this point, an irrefutable case for the existence of PD-replicating sleep disturbances in the rotenone model can simply not be made due to a lack of conclusive findings, transparency in the data presented, inconsistencies between methodologies, and literature support. While some more recent studies have shown promise, a significant and widely-encompassing inquiry into the sleep phenotype of the rotenone PD murine model would be welcome. Further, greater consideration must be given to utilizing a DMSO/PEG vehicle solution, given that DMSO may affect sleep in rats. Thus, whether this model is a suitable PD model in the context of sleep disruption in PD requires further validation studies.

### α-Synuclein Transgenic Models

The aforementioned discovery of α-synuclein as the key component of Parkinsonian intracellular inclusions, and discoveries that mutations in the *SNCA* gene (which encodes α-synuclein) can cause rare autosomal-dominant PD, have led to the development of *SNCA*-targeting murine models [121, 122]. Masliah and colleagues, the pioneers in PD research who described fibril inclusions developed a PD model in which the loss of dopaminergic neurons and subsequent motor deficits developed in mice after overexpressing α-synuclein (referred to as α-synuclein overexpressing, or ASO) [58]. Consequently, and within the last 20 years, there has been a rapid increase in the number of transgenic models of PD.

Today, both wildtype and mutant human α-synuclein variants are employed in murine models of the disease. Of this latter category, the two most frequently used forms are A53T and A30P, which are both associated with early onset familial PD [253]. A variety of promotors have been employed to control the spatiotemporal expression of the transgenes (most notably the mouse *THY1* promoter [59]) and different groups have employed knock-in, bacterial artificial chromosome-transgenic and viral transgenic methods [254]. Although transgenic α-synuclein mouse models exhibit diverse phenotypes, likely due to variations in methodologies, such as the location of the insertion site of the transgenes and promoter efficacy, most models closely recreate the neuropathological features of PD [253].

#### Circadian Rhythm

Circadian investigations in *THY1-ASO* transgenic mice found selective deficits in wheel-running rhythms, including lower active-phase activity and greater fragmentation [255]. Importantly, these changes were progressive and age dependent. Peak and trough expression of the clock gene *PER2* in the SCN did not differ between genotypes and no evidence was found that the photic response of the circadian system was compromised in the ASO mice. Another group observed in the same model an overall suppression in locomotor activity during only the nighttime [61].

Impaired entrainment of the circadian system has also been observed in A53T mice [62]. Activity onset was considerably detached from the onset of darkness in these mice (an advanced phase angle of 2.65 ± 0.5 hours) while in wild-type mice it was tightly coupled to the beginning of the dark phase. In addition to this, re-entrainment following experimental jetlag was compromised. The generation of endogenous circadian rhythm was not affected. While it may seem contradictory that this model has impaired photic entrainment not seen in the aforementioned ASO model, this may be ascribed to the different protocols employed: the former over-expressed normal human α-synuclein under the *THY1* promoter, while in the latter mutant A53T α-synuclein was utilized under control of the murine neuron-specific prion protein promotor (*PrP*). This demonstrates the significant impact the implemented protocol can have on outcomes; diversity in clinical presentation of PD is reflected in the varying phenotypes of ASO animals.

The case supporting the existence of circadian metabolic abnormalities in ASO mice is supported by Cuvelier and colleagues’ experimental evidence that energy expenditure, oxygen consumption and the respiratory exchange ratio are significantly decreased during the light cycle compared to the dark cycle in WT animals, but not in ASO mice [54]. In analyzing these results, it was proposed that they reflected deficits in circadian-regulated behavior.

Integrating these findings, a case cannot be made for the unequivocal presence of circadian deficits in ASO models, however there is compelling evidence that circadian changes are present (Table 1). Further, it is clear that the extent that circadian issues are observed in ASO models is at least somewhat dependent on the specific design of the model itself. While findings that endogenous circadian rhythm generation is not directly affected by the over-expression of α-synuclein protein do not strictly align with findings in human PD patients, compromised entrainment and locomotor behaviors reflect common symptoms of PD. Additional research into the model is required; broadly-encompassing expression profiles of clock genes across the brain would be one investigation which would contribute considerably to a deeper understanding of the circadian features of the model.

#### Sleep

ASO mice exhibit increased NREM sleep during their quiescent phase and decreased REM sleep across both the quiescent and active phases [256]. Further, these mice have pervasive alterations in the EEG power spectra (an increase in delta power and a decrease in alpha power during both NREM and REM sleep bouts, with an additional decrease in theta power during bouts of REM sleep) [256]. Of particular consequence, the EEG profiles of ASO mice shift towards lower frequencies, reminiscent of EEG slowing observed in PD patients (refer to Table 2 in [256] and [63]). Manifestation of these changes considerably predates striatal dopamine loss and motor deficits, which strongly reflects the prodromal sleep changes of PD.

Reports of sleep disturbances in transgenic mice have been supported by analyses of locomotor activity data. Mice which express mutant human α-synuclein have significantly reduced overall sleep time, and elevated horizontal (home cage) activity levels during the resting phase [64]. Temporal distribution of sleep is altered in ASO mutants, with increased sleep latency suggestive of the insomnia experienced by PD patients [255].

Butkovich and colleagues specifically targeted the LC by expressing normal human α-synuclein in noradrenergic neurons [257]. They noted PD-like degeneration on immunohistology, without inclusions, and importantly found a significant increase in sleep latency relative to controls at 3 and 14 months, indicative of an elevated arousal state and analogous to the elevated arousal in prodromal PD. Another similar model in which the LC was targeted found PD-like pathology in neurons and glia, and while the authors explicitly suggested a thorough characterization of sleep should be undertaken, neither they nor other groups have performed this as of publication [258].

These findings, taken collectively, make an argument for the presence of PD-reminiscent sleep dysfunction in α-synuclein transgenic mice (Table 1). While it should be noted that the body of research is somewhat limited, the investigations are complementary and suggest that the model does hold significant promise for investigating both progression of sleep issues in PD, and interplay between sleep disruption and PD pathology. Further corroborating reports would be welcomed to cement the purported phenotype, to justify the use of the α-synuclein model in future investigations of sleep dysfunction in PD.

### VMAT2-Deficient Model

In 2007 Caudle and colleagues developed a potential PD mouse model; a genetic reduction of vesicular monoamine transporter 2 to 5% of normal levels (VMAT2-deficient) resulted in striatal dopamine loss, motor deficits, α-synuclein accumulation, and progressive nigral dopaminergic cell loss [65]. Following this, they released a report on the non-motor symptoms of this model, which included investigations into sleep latency [259]. A shorter latency to behavioral sleep was observed in VMAT2-deficient mice at 2 months of age, which subsequently became more pronounced at 4-6 months of age. While this suggests that sleep disruption does occur, these findings represent a phenotype that is contrary to the insomnia found with high prevalence in Parkinson’s patients. Although reduced amplitude of the sleep-wake cycle was noted in the model, aside from at 4–6 months of age circadian activity of VMAT2-deficient mice was reported to not differ significantly to WT mice.

### MitoPark Model

Dopaminergic neuron-specific deletion of the mitochondrial transcription factor A-encoding gene results in progressive cellular and motor alterations analogous to idiopathic PD in mice: progressive midbrain dopaminergic neuron degeneration and cytoplasmic inclusions of α-synuclein aggregates among others [260]. Termed the ‘MitoPark’ model [261], these mice also recapitulate the impairments to circadian control of sleep-wake cycles widely observed in PD. Age-dependent circadian deterioration, of both rhythm and amplitude, as well as increased sleep fragmentation was noted by Fifel and Cooper [262]. Additionally, it was determined in the same publication that endogenous circadian rhythms are severely perturbed or even abolished in MitoPark mice, evinced by loss of circadian locomotor rhythms in constant light or constant dark conditions. These findings hold great promise for the MitoPark model as a PD model which accurately reflects the circadian and sleep dysfunction features of PD (Table 1).

### SV2 Model

No evidence directly links the synaptic vesicle glycoprotein 2 (SV2) family to PD, although SV2C has emerged as a candidate contributor to PD pathogenesis, as well as being established as a mediator of dopamine homeostasis. Interestingly, SV2C transgenic mice experience disturbed circadian-mediated activity spikes towards the end of the active period, which underlies a reduction in circadian locomotor activity [263]. While SV2C animals aren’t proposed as a PD model, there is the potential that they may reflect some circadian disruption of disease, and as such assist with understanding mechanistic connections between sleep and PD.

### Cycad Model

In rats, consumption of *Cycas micronesica* (Cycad) seeds triggers progressive neurodegeneration, with behavioral, biochemical, and histological hallmarks characteristic of parkinsonism, including α-synuclein aggregates in dopaminergic and noradrenergic neurons of the SNpc and LC [264]. These rats exhibit an increase in length and number of bouts of REM and NREM sleep at the expense of wakefulness during the active period [265]. This hypersomnolent behavior suggests an inability to maintain arousal, similar to the EDS PD patients often present with. Despite this, there were no changes to sleep in the quiescent phase, suggesting that the model doesn’t reflect the full suite of sleep issues faced by PD patients.

### LRRK2 Model

Mutations in the *LRRK2* gene are a key genetic cause of familial PD. It is important to note that the sleep vicissitudes of *LRRK2*-PD patients differ slightly from those of idiopathic PD patients, with increased sleep onset insomnia frequency and less prominent RBD. Further, in *LRRK2*-PD, EDS and RBD are typically absent from the pre-symptomatic stages of the disease [184, 266]. The sleep fragmentation observed in the preclinical murine LRRK2-G2019S model represents one of the four sleep complaints experienced by 56% of LRRK2-PD patients [184, 267]. It remains to be shown whether the sleep fragmentation in these mice is specific for NREM or REM sleep as the study did not differentiate between sleep stages.

## PD Models Uninvestigated for Circadian and Sleep Phenotypes

Given the review concerns the current understanding of sleep and circadian disruptions in murine models of PD, it would be remiss not to briefly discuss models of disease in which these features have not been studied.

### Paraquat/Maneb Model

One such model is the commonly employed paraquat neurotoxic model of PD, which is frequently administered in combination with maneb, a synergistically interacting agent to enhance neurotoxicity [268]. One consideration of this model is that despite the oft-cited structural similarity between paraquat and MPP+, the two have divergent mechanisms of toxicity [60]. Thus, it is important that the validity of the model is adjudged in non-motor contexts, especially as clear motor deficits and striatal dopamine deficiencies are lacking [269].

### Genetic Models

Consideration of circadian rhythms and sleep has been largely excluded in the establishment of several emerging genetic murine models of disease based upon findings of PD-predisposing familial mutations in certain genes other than *LRRK2*, most notably *PINK1*, *PRKN*, *DJ-1*. The circadian phenotypes of drosophila models with homologue knockouts of the latter three genes have been characterized; this is well-reviewed by De Lazarri *et al*. [270]. *PINK1* and *PRKN* flies show a disrupted circadian phenotype [271], while the sole study in *DJ-1* knockout flies did not find evident abnormalities in the circadian rhythmicity. No investigations have been undertaken in these murine models targeting these genes.

### PFF Model

The pre-formed fibril (PFF) PD seeding model, first proposed by the Trojanowski-Lee lab in their pioneering 2012 Luk *et al.* publication [272], has been increasingly found to accurately and robustly model Parkinsonian symptomology and pathology. The model is comprised of wildtype non-transgenic mice inoculated intracerebrally with α-synuclein PFFs, which are subsequently transmitted in a prionoid fashion from cell to cell. As a result, a time-dependent spread of cellular pathology *via* the neural connectome is seen; progressive nigrostriatal neuron death and motor deficiencies occur consequent to this. While the model is increasingly being utilized within the PD research community, there remains a lack of research as to the whether the PFF model has sleep and circadian phenotypes resembling the sleep disturbances of human PD (Table 1). Notably, one assumes that the site of inoculation largely determines the spreading, sites of pathology and sequence of behavioral changes.

## Conclusions

Research increasingly indicates that sleep dysfunction and circadian arrhythmicity are key aspects to consider when investigating neurodegenerative disease, particularly in PD. Given this, while recognizing that there are numerous inter-species differences in circadian rhythms and sleep which poses a significant issue in interpreting the relevance of any changes in murine models of PD, it is important to enlist murine models which replicate the PD symptom spectrum as accurately as possible. Sleep dysfunction in PD has significant negative impacts on both patient and carer quality of life; due to improved motor symptom management capability, management of circadian dysfunction and sleep disorders is to become a progressively more central facet of PD care. There is a dual necessity to consider sleep in disease: 1) to enable development of circadian-facing therapeutic treatments and, 2) to investigate mechanistic interplay between sleep and disease progression. When investigating any affliction, it is essential to use models which most accurately reflect the disease, and as such, with sleep disorders one of the most prominent non-motor symptoms of disease [273], it must be a key consideration of PD research. Equally important is to use preclinical animal models that closely reflect human circadian physiology, for example a mouse strain that is pineal melatonin proficient (e.g., C3H/HeN) as opposed to pineal melatonin-deficient mice commonly used in Neuroscience research (e.g., C57BL/6). As noted towards the beginning of this review, melatonin rhythm dysfunction, including decreased melatonin levels, is associated with PD, and it is often utilized in PD treatment, particularly for PD-associated sleep dysfunction. Interestingly, the quality of the evidence on which melatonin treatment of sleep disturbances in PD is based is suboptimal. A detailed clinical digest on this topic can be found here [150]. On the other hand, many studies in mouse and rat models have investigated whether melatonin may play a neuroprotective, anti-inflammatory, and antioxidant role, administering melatonin prior or subsequent to chemical insult or seeding, with largely positive outcomes (see Table 1 in [274]). However, there appears to be a relative dearth of investigations into whether endogenous melatonin rhythms are affected in models; this is potentially also due to studies being performed in pineal melatonin-deficient mice (e.g., [255, 275]). We suggest that melatonin rhythms should be characterized in the current and future models where possible, and that studies investigating a therapeutic role for melatonin should attempt to use models which replicate melatonin rhythm dysfunction seen in human PD.

Hence, we have here characterized the current state-of-the-art in terms of rodent models of PD and literature regarding their associated sleep and circadian rhythm features. From this, we have been able to reflect upon the accuracy of these models with disease, as well as highlight significant areas and/or models which are requiring further investigations. A summary of these findings can be found in Table 1. The 6-OHDA and α-synuclein transgenic models reflect the sleep and circadian rhythm dysfunction associated with PD most accurately, provided current results are further corroborated. The MitoPark model of disease shows significant promise, however further investigation is required to confirm this to a greater extent. Murine rotenone-PD models display some aspects of PD-like sleep defects; the literature is inconclusive, and no complete consensus can be drawn. The MPTP model of disease does not reflect the sleep and circadian rhythm dysfunction of PD, and as such this is a major caveat when considering disease investigations employing this model. The cycad model, while severely limited in terms of breadth of research, is also unlikely to be a good model of PD-like sleep dysfunction. There are a number of models which are yet to be investigated fully, including the PFF and paraquat/maneb models, as well as a number of transgenic models.

In terms of future directions, it is important that these disease models are characterized more comprehensively in terms of circadian and sleep features. Sleep and circadian rhythm dysfunction presents near ubiquitously in the clinical setting, and there is a need for further detailed investigation of circadian alterations and sleep disorders in PD, utilizing modern research methodologies and tools, to elicit insight into both mechanisms of disease progression and potential therapeutic treatments [2, 3, 15–17]. We recognize that delving into the specifics of this would require an entire focused review on just that topic, so highlight the overall need rather than attempting to summarize the explicit considerations here. While several models have been investigated by a wide array of groups, it would be ideal if there could be a standardized set of experiments and measurements to allow comparisons between disease models, especially in emerging transgenic models. It is noted that different models of disease may be used for different investigations into disease and as such may not always be directly comparable, however setting out these strengths and weaknesses of models is an important part of the process of refining disease models as new information comes to light, which is also in part the aim of this review with respect to sleep and circadian phenotypes.

Based on the presented findings, we must re (evaluate) the purpose of the current PD models in terms of further Parkinson’s investigations. One key point to be made here is that the neurotoxic models, where chemical lesioning induces neurodegeneration and Parkinson’s symptoms, investigations are limited to symptom management and potential neurogenesis, and not suitable to explore the underlying mechanisms which lead to human PD development. In the particular context of sleep and circadian rhythm dysfunction, it can only be seen as to the effects that degeneration in certain brain regions have on sleep and circadian rhythms. On the other hand, in PFF and transgenic models, which offer a more accurate recapitulation of the progressive nature of the disease, the interface and interplay between sleep and degeneration can be investigated, as well as effects of potential therapeutic treatments on disease progression. This difference is particularly stark when considering that sleep disruption frequently appears prior to the emergence of motor symptoms, which is a phenomenon unable to be investigated in neurotoxic models. For this reason, especially in the context of sleep and circadian rhythm aspects of PD, the authors believe that transgenic models of disease will become of far greater importance to understanding these mechanisms. This is part of the reason that these models must be more comprehensively categorized to assist in unravelling this two-way interplay between disease progression and sleep defects.

## Data Availability

Not applicable

## Abbreviations

PD: Parkinson’s disease
HD: Huntington’s disease
AD: Alzheimer’s disease
SCN: Suprachiasmatic nucleus
TTFL: transcriptional-translational feedback loop
CLOCK: Circadian Locomotor Output Cycles Kaput
NPAS2: Neuronal PAS Domain Protein 2
BMAL1: Muscle ARNT-Like 1
Cry1: Cryptochrome 1
Cry2: Cryptochrome 2
Per1: Period 1
Per2: Period 2
ROR: Retinoic Acid-Related Orphan Receptors
ipRGCs: intrinsically photosensitive retinal ganglion cells
VLPO: ventrolateral preoptic area
LC: locus coeruleus
DMH: dorsomedial hypothalamus
LH: lateral hypothalamus
REM: rapid eye movement
NREM: non-rapid eye movement sleep
EMG: electromyography
EEG: electroencephalography
SWS: slow-wave sleep
SNpc: substantia nigra pars compacta
DA: dopamine
SNCA: SyNuClein Alpha gene that codes for the αS protein
LRRK2: leucin-rich repeat kinase 2
PARK7: parkinson protein 7
PINK1: PTEN-induced kinase 1
PRKN: parkin
CBT: core body temperature
L-Dopa: levadopa, a dopamine precursor to treat PD symptoms
RLS: Restless legs syndrome
EDS: excessive daytime sleepiness
6-OHDA: 6-hydroxydopamine
ROS: reactive oxygen species
VTA: ventral tegmental area
SS: sleep spindle
MPTP: 1-methyl-4-phenyl-1,2,3,6-tetrahydropyridine
DMSO: dimethyl sulfoxide
PEG: polyethylene glycol
ASO: α-synuclein overexpressing
PrP: prion protein promotor
WT: wildtype
VMAT2: vesicular monoamine transporter 2
SV2: synaptic vesicle glycoprotein 2
PFF: pre-formed fibril
